# COVID-19 among workers assisting homeless and socially vulnerable people

**DOI:** 10.31744/einstein_journal/2022AO6237

**Published:** 2022-03-04

**Authors:** Nídia Celeste Horie, Karin Schmid, Brayan Filipe Farias da Silva

**Affiliations:** 1 Serviço Franciscano de Solidariedade Província Franciscana da Imaculada Conceição do Brasil São Paulo SP Brazil Serviço Franciscano de Solidariedade, Província Franciscana da Imaculada Conceição do Brasil, São Paulo, SP, Brazil.

**Keywords:** COVID-19, Coronavirus infections, Homeless persons, Communicable diseases, Occupational risks, SARS-CoV-2, Health care (Public health, Vulnerable populations, Occupational health, Infectious disease transmission, patient-to-professional

## Abstract

**Objective:**

To describe the profile of professionals assisting homeless and socially vulnerable populations tested for COVID-19, and to determine potential associations with exposure at the workplace, on the way to work, or at home, among infected professionals. To describe disease symptoms and progression and to investigate potential associations with age, sex and exposure at the workplace, on the way to work, or at home.

**Methods:**

A retrospective analysis of data of 173 workers employed by *Serviço Franciscano de Solidariedade* tested for SARS-CoV-2. Between May 20 and June 2, 2020, professionals and volunteers were tested for anti-SARS-CoV-2 IgG and IgM antibodies, by means of qualitative rapid chromatographic immunoassay in whole blood. A questionnaire was used to collect data on demographic characteristics and working conditions, history and date of onset of symptoms and risk factors. Quantitative variables were expressed as mean and standard deviation, or median, maximum, and minimum values. Data normality was investigated using the Kolmogorov-Smirnov test.

**Results:**

A total of 46 (26.6%) participants had positive serologic tests. Of participants with negative serologic test results, 109 (85.8%) were asymptomatic. History of symptoms was the most significant independent factor associated with positive serology. Serologic test results and symptoms differed significantly according to housing (p=0.045) and working (p<0.001) conditions. More than half of participants (52.4%) living in shared households tested positive, compared to 23% of participants living in family households. Participants working remotely from home did not test positive. In seropositive participants, onset of symptoms was associated with workplace exposure and shared housing conditions.

**Conclusion:**

History of symptoms was associated with positive serology for COVID-19. Shared housing conditions tended to be associated with higher risk of infection. Onset of symptoms was associated with higher levels of workplace exposure and shared housing conditions in seropositive participants.

## INTRODUCTION

In December 2019, news started to spread about a new type of pneumonia detected in Wuhan, China. On January 7, 2020, Chinese authorities informed the disease was caused by a novel coronavirus, which was named severe acute respiratory syndrome coronavirus 2 (SARS-CoV-2) and the resultant disease was later described as coronavirus disease 2019 (COVID-19).^(1)^ In Brazil, a total of 6,730,118 cases had been reported and 179,032 deaths confirmed up to December 10, 2020.^(2)^

The homeless are one of the most vulnerable social groups.^(3)^ Chronic, uncontrolled conditions, such as alcoholism, respiratory diseases, malnutrition and psychiatric disorders affect approximately 70% of homeless people. In the United Kingdom, the average lifespan of a homeless person is 47 years, due to comorbidities and violence-related reasons.^(4)^ Homeless people have limited access to isolation and basic hygiene measures; therefore, they are less protected from infectious diseases.

The 2019 Census on Homeless People living in the city of São Paulo (SP) counted 24,344 individuals, and over 100 thousand people are estimated to be in this situation in Brazil.^(5)^

Efforts to protect homeless people during the COVID-19 epidemic have been described in some countries, such as the United States, Singapore and Malaysia.^(6)^ In Boston (U.S.A.), a structured program aimed to assist homeless people reported 33.1% (429 individuals) of positive test results in this population, within 6 weeks, indicating a high number of cases in shelters.^(7)^

Professionals providing assistance to this population must be protected. According to surveys with health care professionals, area of work, types of personal protection equipment, profession and community exposure are risk factors for COVID-19 infection.^(8-11)^ According to a study investigating tuberculosis transmission among professionals assisting homeless people in Montreal, Canada, these individuals were at an even higher risk of infection than health care professionals.^(12)^

*Serviço Franciscano de Solidariedade* (SEFRAS) is a not-for-profit social organization providing care to more than 3,000 people on a daily basis, including children, youngsters, older adults, homeless and immigrants. Since March 2020, SEFRAS acts primarily in provision of food to homeless and socially vulnerable people.

SEFRAS designed a plan to tackle coronavirus (*Plano de Enfrentamento ao Coronavírus*) describing specific preventive measures recommended for protection of the target population and collaborators.^(13)^ As to use of personal protection equipment (PPE) during activities involving direct contact with people, face masks have been recommended since March 19, 2020, with other precautions following. Laboratory aprons, glasses, caps and gloves were later included in the PPE list for these professionals. Since April 16, 2020, use of face mask was implemented in all working spaces at SEFRAS. As from May 7, 2020, use of face mask in public spaces has been compulsory in the state of São Paulo.

No articles describing COVID-19 infections among professionals assisting homeless and socially vulnerable populations in Brazil have been published to date, nor has the efficacy of protective measures been described.

Professionals with higher levels of workplace exposure are likely to be at increased risk of infection. However, the use of PPE may be enough and appropriate to prevent infection.

Household transmission may be a relevant factor among people living in shared housing conditions. Use of public transportation may also impact the risk of infection.

## OBJECTIVE

To describe the profile of professionals assisting homeless and socially vulnerable populations who were tested for COVID-19, and to determine potential associations with exposure at the workplace, on the way to work, or at home, among infected professionals. To describe disease symptoms and progression, and to investigate potential associations with age, sex and exposure at the workplace, on the way to work, or at home.

## METHODS

This is a retrospective, cross-sectional, quantitative study. Data were collected from professionals and volunteers working at SEFRAS, in the city of São Paulo. Given the lack of tests for COVID-19 at the time, as of early March 2020, professionals with suspected infection were isolated and referred to health care services. Between May 20 and June 2, 2020, all professionals and volunteers were tested for anti-SARS-CoV-2 immunoglobulins G and M (IgG and IgM, respectively) antibodies, using qualitative rapid chromatographic immunoassay in whole blood (Hi Technologies, Hilab, Curitiba, PR, Brazil). They also answered questionnaires inquiring into demographic data, place of work, history and date of onset of symptoms, and risk factors. According to manufacturers, test sensitivity and specificity for IgG and IgM were as follows: 100% sensitivity and 98% specificity for IgG, and 85% sensitivity and 96% specificity for IgM.^(14)^ Test results were considered positive whenever IgG or IgM positivity was detected.

Participants were divided into four categories according to levels of workplace exposure: home office - working remotely from home, or visiting the workplace no more than once a week; no contact - workers involved in administrative or internal activities, with no direct contact with the public; limited contact - workers performing in-person activities involving direct contact with the public for no more than 1 hour and 30 minutes per day; high contact - workers performing in-person activities involving direct contact with the public most of the time.

As to living conditions, participants were classified as follows: family households shared with up to nine people belonging to the same family, and collective households shared between ten and 50 people. Participants were also classified as public transportation users or not.

Participants who tested positive also answered a questionnaire about severity of symptoms and were classified as asymptomatic, mild symptoms (had symptoms but were able to work or perform household chores), or moderate symptoms (bedridden at home or requiring hospital admission with no need of intensive care).

Quantitative variables were expressed as mean and standard deviation, or median, maximum and minimum values. Data normality was investigated using the Kolmogorov-Smirnov test, followed by the examination of normal probability plots for sample distribution assessment. Continuous variables were compared between symptomatic or asymptomatic participants with positive or negative serologic test results using analysis of variance (ANOVA) or the Kruskal-Wallis test, as appropriate. Associations between qualitative variables were tested using the Pearson’s χ^2^ test or the likelihood ratio, as appropriate.

Non-adjusted odd ratios (OR) and respective 95% confidence intervals were estimated for each of the characteristics of interest using simple logistic regression. Adjusted OR were estimated using multiple logistic regression applied to the model containing all variables of interest (adjusted analysis) and retaining all variables in the final model (full model). The level of significance was set at α=0.05.

All participants signed an informed consent term. This study was approved by the Ethics Committee of *Universidade São Francisco* (USF). This project was submitted to and approved with CAAE: 34538420.0.0000.5514, opinion number 4.149.135.

## RESULTS

**Figure 1:**
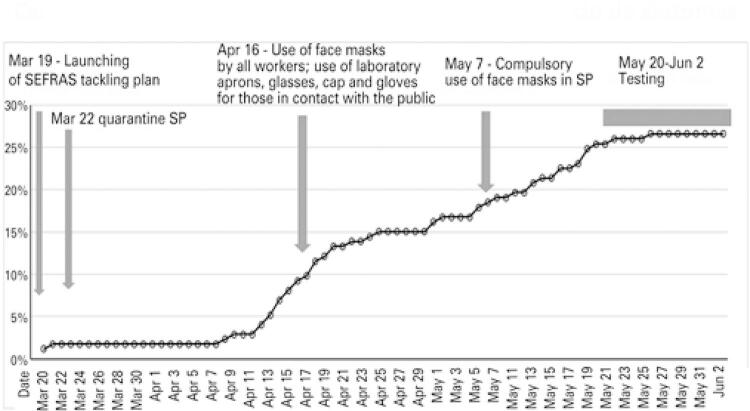
Cumulative number of new COVID-19 cases in 2020 according to date of onset of symptoms

The sample comprised 173 workers, mean age 38.6 (10.5) years. Of these, 99 (57.2%) were women and 46 (26.6%) tested positive for SARS-CoV-2. Characteristics of symptomatic or asymptomatic participants with positive or negative test results are listed in table 1. The progression of positive cases over time is shown in figure 1. Twenty-six male and 20 female participants (35.1% and 20.2%, respectively) had positive serologic test results. Only 18 (10.4%) participants lived by themselves and 21 (12.1%) shared a household with more than nine people. More than half of participants (52.4%) living in shared housing conditions had positive serologic test results, compared to 23% of participants living in family households. Serologic test results also differed according to working conditions, with no positive results among participants working remotely from home.

**Table 1:** Characteristics of participants according to symptoms and SARS-CoV-2 serologic test results

Characteristics	Negative serology		Positive serology		p value
	
	Asymptomatic	Symptomatic	Asymptomatic	Symptomatic	
n (%)	109 (63.0)	18 (10.4)	9 (5.2)	37 (21.4)	
Age, mean±SD	39.3±10.7	35±7.3	42.4±9.1	37.7±11.2	0.257
Sex, n (%)					0.152
Male, n=74	41 (55.4)	7 (9.5)	6 (8.1)	20 (27.0)	
Female, n=99	68 (68.7)	11 (11.1)	3 (3.0)	17 (17.2)	
Cohabitants	3 (1-40)	3 (1-15)	5 (2-30)	3 (1-50)	0.180
Housing, n (%)					
Family, n=152	101 (66.4)	16 (10.5)	6 (3.9)	29 (19.1)	0.045
Shared, n=21	8 (38.1)	2 (9.5)	3 (6.4)	8 (17.3)	
Public transportation, n (%)					
Yes, n=110	73 (66.4)	11 (10.0)	7 (6.4)	19 (17.3)	0.290
No, n=63	36 (57.1)	7 (11.1)	2 (3.2)	18 (28.6)	
Working conditions, n (%)					
Home office, n=33	27 (81.8)	6 (18.2)	0	0	<0.001
No contact, n=48	33 (68.8)	1 (2.1)	3 (6.3)	11 (22.9)	
Limited contact, n=45	25 (55.6)	5 (11.1)	6 (13.3)	9 (20.0)	
Much contact, n=47	24 (51.1)	6 (12.8)	0	17 (36.2)	

Results expressed as n (%) or median (minimum-maximum value).

SD: standard deviation.

SEFRAS: *Serviço Franciscano de Solidariedade.*

A total of 18 workers had symptoms consistent with COVID-19. Of these, 15 reported fever and had negative serologic test results (14.2% of SARS-CoV-2 negative participants). None of these participants were submitted to reverse transcription polymerase chain reaction (RT-PCR) test for SARS-CoV-2 at the onset of symptoms or tested for influenza. None of them required hospital admission.

Findings of adjusted logistic regression analysis of variables associated with positive serologic test results are shown in table 2.

**Table 2:** Logistic regression analysis of associations between variables and positive SARS-CoV-2 serologic test results

Variable	Non-adjusted			Adjusted		
	
	OR	95%CI	p value	OR	95%CI	p value
Male sex	2.14	1.08-4.24	0.029	2.25	0.86-5.92	0.100
Age, years	1.00	0.97-1.03	0.977	1.04	1.00-1.09	0.069
Working conditions						
Home office	*		0.998			
No contact	0.77	0.33-1.83	0.558			
Limited contact	0.88	0.37-2.08	0.775			
High contact	References		References			
Shared housing	3.68	1.44-9.38	0.006	3.96	0.99-15.94	0.052
Public transportation	0.67	0.33-1.33	0.247	1.44	0.53-3.93	0.474
Symptoms	24.90	10.30-60.19	<0.001	32.89	12.05-89.78	<0.001

* No cases to estimate.

OR: odds ratio; 95%CI: 95% confidence interval.

The variables “work condition” and “housing condition” were highly collinear (*i.e.*, participants with higher levels of workplace exposure also tended to live in collective households). Therefore, the variable “work condition” was excluded from the adjusted model (Table 3).

**Table 3:** Cross-tabulation between working and housing conditions among workers employed by *Serviço Franciscano de Solidariedade*, 2020

Working conditions	Housing conditions			p value*

	Family n (%)	Shared n (%)	Total	
Home office	35 (100.0)	0	35	<0.001
No contact	44 (95.7)	2 (4.3)	46	
Low contact	37 (82.2)	8 (17.8)	45	
High contact	36 (76.6)	11 (23.4)	47	
Total	152 (87.9)	21 (12.1)	173	

* χ^2^ test for trend.

Following model adjustment, “manifestation of symptoms” was the only variable associated with seropositivity. However, shared housing also tended to be associated with seropositivity (adjusted OR 3.96; p=0.052).

**Figure 2:**
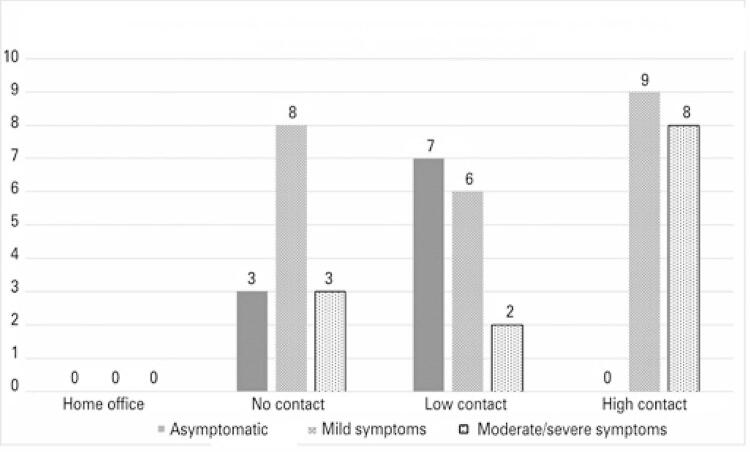
Manifestation and severity of symptoms in participants with positive SARS-CoV-2 serologic test results, according to working conditions

The distribution of participants with positive serology according to manifestation of symptoms or lack thereof is shown in table 4. Working conditions appeared to impact the severity of symptoms, given all workers with high levels of workplace exposure were symptomatic. Of these, eight (57.1%) had moderate symptoms, compared with only three (21.4%) participants with moderate symptoms among those with no workplace exposure (Figure 2). Two out of eight participants with moderate symptoms were hospitalized. However, none of them required intensive care or mechanical ventilation, or progressed to death.

**Table 4:** Characteristics of participants according to symptom classification

Characteristics	Symptoms			p value

	Asymptomatic	Mild symptoms	Moderate symptoms	
n (%)	10 (21.7)	23 (50.0)	13 (28.3)	
Age, mean±SD	41.1±9.6	39.9±10.7	39.7±12.4	0.557
Sex, n (%)				0.199
Male, n=26	7 (26.9)	10 (38.5)	9 (34.6)	
Female, n=20	3 (15.0)	13 (65.0)	4 (20.0)	
Comorbidity, n (%)				0.684
Yes, n=8	1 (12.5)	5 (62.5)	2 (25.0)	
No, n=38	9 (23.7)	18 (47.4)	11 (28.9)	
Working conditions, n (%)				0.006
No contact, n=14	3 (21.4)	8 (57.1)	3 (21.4)	
Low contact, n=15	7 (46.7)	6 (40.0)	2 (13.3)	
High contact, n=17	0	9 (52.9)	8 (47.1)	
Public transportation, n (%)				0.514
Yes, n=20	3 (15)	10 (50.0)	7 (35.0)	
No, n=26	7 (26.9)	13 (50.0)	6 (23.1)	
Housing, n (%)				0.044
Family, n=35	6 (17.1)	21 (60)	8 (22.9)	
Shared, n=11	4 (36.4)	2 (18.2)	5 (45.5)	

SD: standard deviation.

Only eight (17.4%) participants with positive serology reported prior comorbidities. Comorbidities were not associated with severity of symptoms. Comorbidities reported were hypertension, type 2 diabetes, heart disease and chronic lung disease (four, two, one, and one participant, respectively).

## DISCUSSION

This study was based on qualitative rapid chromatographic immunoassay test results. This was a major limitation. In spite of good levels of sensitivity and specificity reported by test manufacturers, the role of serology in COVID-19 diagnosis has been disputed due to the low accuracy of some tests.^(15)^ Nose swab RT-PCR is the current standard for COVID-19 diagnosis. However, participants of this study did not have access to this test. Also, many could only be tested 2 weeks after the onset of symptoms, when RT-PCR tests are no longer applicable. In this sample, 18 participants with negative serology developed symptoms. This may have been partially due to other, self-limiting infections, or to non-infectious conditions, such as myalgia and headache. These conditions were more strictly surveilled. Still, should those be false-negative (18/64), this sample would have comprised 28.1% of false-negative, which is in keeping with the number of false-negative in nasal swab RT-PCR tests.^(16)^ At the same time, given most participants (78.3%) were symptomatic, it is unlikely this sample comprised a high number of false-positive. Higher frequency of positive test results among asymptomatic patients has been reported^(7)^

In this study, participants working remotely from home or visiting the workplace no more than once a week were the ones with the lowest risk of workplace exposure. The fact that none of these workers had positive serologic test results supports the efficacy of isolation measures. The number of cases with moderate symptoms was also higher (47.1%) among participants with higher levels of workplace exposure, with no asymptomatic cases detected in this group. Only five (17.2%) of participants with no or low levels of workplace exposure developed moderate symptoms. One study involving 198 countries revealed lower mortality rates in those adopting face mask use policies or rules.^(17)^ The premise that viral load is related to the odds of infection and disease severity has been tested for influenza. More severe symptoms were associated with higher viral loads among healthy volunteers who received varying doses of influenza virus.^(18)^

Given the mode of transmission of COVID-19, it would be reasonable to expect a higher risk of infection among users of public transportation, as in the case of influenza.^(19)^ This risk has been reported in studies investigating COVID-19 spread.^(20)^ Studies investigating social distancing measures in 149 countries (school and workplace closure, public transport shutdown, and meetings, public events and movement restrictions) also revealed a significant impact of these measures on the incidence of COVID-19.^(21)^ However, public transport shutdown appeared to have no additional benefits regarding risk mitigation, provided other social distancing measures were in place. In this sample, use of public transportation was not associated with higher risk of positive serologic test results. Implementation of risk mitigation measures (use of face masks and opened windows) in public transport networks may have offset exposure.

In this sample, living in households shared with large numbers of people tended to be associated with positive serologic test results and manifestation of symptoms. High density housing conditions are associated with greater social vulnerability and higher risk of infectious disease transmission.^(22)^ Moreover, non-use to PPE was detected even in low density households in this study, facilitating infection.

As to working conditions, home office was the most significant factor. Although professionals with higher levels of workplace exposure were thought to be at greater risk of infection, these workers were also the first to adhere to PPE use. Also, protective measures to prevent transmission among workers with no contact with the public were reinforced in early April, after that the new case curve became less steep.

## CONCLUSION

History of symptoms was the most significant factor associated with positive serology among workers and volunteers assisting homeless and socially vulnerable populations during the COVID-19 pandemic. Risk of COVID-19 infection tended to be associated with shared housing conditions. In infected participants, symptom development was associated with higher levels of workplace exposure and shared housing conditions. Adoption of protective measures in the workplace and in urban areas appeared to translate into a drop in the number of new cases, supporting the need of such measures to mitigate the risk of infection.
